# A New LC-MS/MS Method for Simultaneous and Quantitative Detection of Bisphenol-A and Steroids in Target Tissues: A Power Tool to Characterize the Interference of Bisphenol-A Exposure on Steroid Levels

**DOI:** 10.3390/molecules25010048

**Published:** 2019-12-21

**Authors:** Sonia Errico, Teresa Chioccarelli, Martina Moggio, Nadia Diano, Gilda Cobellis

**Affiliations:** Department of Experimental Medicine, University of Campania “L. Vanvitelli”,Via S. M. di Costantinopoli, 16, 80138 Naples, Italy; sonia.errico@unicampania.it (S.E.); teresa.chioccarelli@unicampania.it (T.C.); ma.moggio@studenti.unina.it (M.M.); gilda.cobellis@unicampania.it (G.C.)

**Keywords:** Bisphenol A, 17β-estradiol, testosterone, LC-MS/MS, testis, visceral fat

## Abstract

Bisphenol A (BPA), an endocrine disruptor, may affect in situ steroidogenesis and alter steroids levels. The present work proposes a liquid chromatography tandem mass spectrometry method to simultaneously quantify BPA, 17β-Estradiol and testosterone in two target tissues: testis and visceral fat mass. Analytes were isolated and lipophilic impurities removed by two serial steps: liquid-liquid and solid phase extraction. All compounds were separated in a single gradient run by Kinetex F5 column and detected via multiple reaction monitoring using a triple quadrupole with a TurboIon electrospray source in both negative and positive modes. The method is selective and very sensitive. In the investigated concentration range, the linearity of the detector response is verified in both tissues. The use of specific SPE cartridges for affinity chromatography purification allows obtaining high percentages of process efficiency (68.0–83.3% for testicular tissue; 63.7–70.7% for visceral fat mass). Good repeatability and reproducibility was observed. The validated method can be efficiently applied for direct biological monitoring in testis and visceral fat mass from mice exposed to BPA. The quantification of compounds in a single assay could be achieved without a loss of sensitivity.

## 1. Introduction

Bisphenol A (BPA), 17β-Estradiol, and testosterone are three chemicals with different origins and functions but linked by their similar structure. BPA is a synthetic organic pollutant used as a common ingredient in the manufacture of polycarbonate plastics or epoxy resins [1]. For instance, 17β-Estradiol (E2) and testosterone (TT) are steroid hormones which control the development and maintenance of secondary sex characteristics and reproductive functions [2]. They play a critical role in many physiological processes, such as bone and lipid metabolism, glucose homeostasis, growth, and cardiovascular function [3,4,5,6].

It is well known that BPA acts competitively toward endogenous hormones considering its capacity to bind several receptors [7,8,9,10], but it can also alter in situ steroidogenesis [11,12] and interfere with the expression and activity of steroidogenic enzymes [13,14,15].

Exposure to BPA even in minute albeit repetitive dose has adverse tissue-specific effects, exerting a non-monotonic dose response [16,17]. Consequently, reliable and accurate measurements of this chemical are crucial to explore the possible molecular mechanisms of action of BPA in target tissues, and to develop interpretative models able to correlate the presence of this environmental pollutant to physiological alterations in organisms exposed to it.

Here, we report an accurate and sensitive liquid chromatography tandem mass spectrometry (LC-MS/MS) assay for the simultaneous measurement of BPA, E2, and TT in two tissues: testis and visceral fat mass. The testis is the primary organ responsible for production of TT and E2. The adipose tissue is a primary tissue into which lipophilic contaminants such as BPA can accumulate [18,19,20], and wherein the biosynthesis of estrogens via peripheral aromatization predominantly occurs [21].

Several studies have investigated the association between exposure to environmental BPA and steroid levels but the data were obtained by measuring BPA concentrations in urine while E2 and TT concentrations in plasma [22,23,24]. To our knowledge, there are no methods to determine with a single analysis the concentrations of the three analytes in biological tissues, particularly the testis and visceral fat.

E2 and TT levels are usually measured in plasma by radioimmunoassays or enzyme-linked immunoassays to evaluate reproductive status and function, to diagnose infertility, delayed or precocious puberty, and other diseases related to altered steroid hormone metabolism [25,26,27,28,29]. Most of the analytical methods measure the concentration of the single analyte with high level of inaccuracy, lack of sensitivity, especially at the low values commonly observed for estradiol in men and postmenopausal women, and testosterone in women, children and hypogonadal men [30,31,32,33,34]. Liquid chromatographic methods connected to mass spectrometry are more specific, eliminating possible interference caused by other endogenous and exogenous compounds affecting the three immunoassays [35,36,37] and obtaining the unambiguous identification of analogous compounds [38]. Mass spectrometry (MS) identifies organic molecules according to their molecular mass and allows their detection and quantification with extremely high sensitivity. Solid phase extraction (SPE) on molecularly imprinted polymer (MIP) cartridges permits a simultaneous extraction of analytes, clean-up, and concentration of the sample, improving the selectivity due to the presence of specific sites for the binding of the analytes, reducing the presence of matrix components, and consequently increasing method sensitivity [39,40]. Used in tandem, the two methodologies yield rapid, cost-effective and quantitative measurements of organic molecules, finding wide applications specially in food analysis and in environmental monitoring [36,41,42]. Our challenge was to use them (i.e., SPE coupled to MS) to develop a new method to simultaneously quantify BPA, E2 and TT in target biological tissues, complex matrices for analytical chemistry.

The proposed method has been validated attending the following criteria: linearity, limits of detection (LOD) and quantification (LOQ), process efficiency, precision, and accuracy. Then, it has been applied for direct biological monitoring in testis and visceral fat (vFAT) mass from mice exposed to BPA. The quantification of compounds in a single assay has been achieved without a loss of sensitivity.

## 2. Results

### 2.1. Method Validation

The developed method is selective. A Phenomenex Kinetex F5 reversed phase column (100 × 4.6 mm, 2.6 μm) produced adequate analyte retention times and separation of compounds, instead of a C18 column. Various mobile phase configurations, flow rates, and profiles were evaluated. The desired sensitivity was achieved by using water and methanol as the aqueous and organic solvents, respectively, under linear gradient elution. The analytes were loaded on the analytical column in 30% mobile phase A and eluted with 95% mobile phase B (see Section 3.6). The optimal chromatographic conditions were flow rate 300 μL min^−1^ and injection volume of 20 μL. The optimized conditions have allowed for the identification of the analytes by retention times: 7.3 min for BPA, 8.0 min for E2, and 8.6 min for TT (Table 1).

The best potential values for DP, EP, and collision gas flow were obtained by direct infusion of the standards switching negative/positive ESI polarity to enable the highly sensitive analysis of all analytes within a single MS assay. For better yield, negative ESI was chosen for BPA and E2 ion transitions and positive ionization for TT. The transitions monitored in MRM mode for BPA were 227.1 > 212.1 and 227.1 > 133.2 *m*/*z* for its quantifier and qualifier ions, respectively, and 243.1 > 215.0 and 243.1 > 132.1 *m*/*z* for its internal standard. The transitions monitored in MRM mode for 4 E2 were 271.0 > 145.0 and 271.0 > 183.0 *m*/*z* for its quantifier and qualifier ions, respectively; for TT were 288.9 > 97.1 and 288.9. > 109.2 *m*/*z* for its quantifier and qualifier ions, respectively. The optimized ESI and MS/MS parameters, including retention times and MRM conditions, were summarized in Table 1.

The method is very sensitive. In the investigated concentration range (0.1–100 ng/mL), the calibration curves, obtained by analyzing in triplicate five standard concentrations, were linear (regression coefficient equal to 0.991 or better). The Table 2 shows the values of slope, intercept, and coefficient of determination relative to a single analyte in the different tissues. Also, the limit of detection (LOD) and the limit of quantification (LOQ) were reported. The slopes of calibration curves by standards prepared in target tissues differed at most by 4% from the slopes of calibration curves in water/methanol (matrix free), difference expressed as coefficient of variation (CV%). These results indicated there was no ion suppression or enhancement and the method was minimally affected by different matrices [43]. These results are in agreement with those findings reported in the literature [44] which establish that matrix effects are minor or eliminated when using LC-MS/MS. For this reason, the calibration curves were prepared in water/methanol and repeated after each set of samples.

The extraction procedure exhibited high specificity. Coupling liquid-liquid extraction (LLE) to solid phase extraction (SPE), the method was markedly selective, especially for adipose tissue, improving the consistency of chromatographic separation and prolonging chromatographic column lifetime. During the sample preparation, the lipid extract from the LLE contains many no polar compounds, such as fatty acids and phospholipids. These lipids can accumulate on the analytical columns, deteriorating the separation and providing a source of ion suppression and matrix effects in LC-MS/MS analyses [45]. Performing SPE using specific cartridges facilitates the removal of interfering compounds, owing to the molecularly imprinted stationary phase, specific for steroids but effective for multi-residue purification, of structurally related compounds such as BPA. In the chromatograms, a lower baseline noise was observed, and no interfering peaks from the matrices were detected, proving effective sample clean-up and no co-eluting endogenous substances that could influence the ionization of the analytes.

The extraction process is efficient. By using a practical and experimental approach proposed by Matuszewski et al. [46] to assess together matrix effect and recovery (see Section 3.7.3), the process efficiency was calculated. In addition to the bioanalytical validation guidelines, by applying this methodology at three levels of concentration simultaneously with the accuracy and precision studies, the number of experiments to perform was minimized and at the same time required information was obtained. The percentage values reported in Table 2 ranged from 68.0% to 83.3% for five testicular tissue, from 63.7 to 70.7% for vFAT mass. The results confirmed the vFAT is a more complex matrix to analyse, but the proposed method based on liquid–liquid coupled with solid phase extraction was able to overcome matrix-related limits. In addition, the efficiency resulted very good for higher concentration levels, highlighting the possibility of using a single assay even for high analyte concentrations without the risk of saturating the cartridges and loss of sensitivity. The method is precise. The intra-day precision, expressed as RSD, was proven to be equal to or lower than 15%, whereas inter-day precision was maximum 18% (Table 3). Accuracy, determined by comparing the mean result for five analyses to the nominal concentration value, was between 85.6 and 104.3% at all concentration levels (Table 3).

To archive the matrix-independence of measures and to compensate losses during sample preparation, isotope-labeled internal standard was used in this study. In particular only BPA-d_16_ was chosen considering that the three analytes have a very similar structure. Conventional analytical methods commonly use internal standards or surrogates to achieve accuracy. Notwithstanding, they are time consuming and increase the cost of the analysis. Furthermore, this methodology included parameters to evaluate process efficiency, matrix effect, and ion interference and an acceptable accuracy was achieved.

### 2.2. Application of the Validated Method

The results demonstrated that the analytical procedure developed in this study is an effective and reliable method to extract and quantize BPA and steroids in complex matrices, such as testis and vFAT mass.

To validate the experimental procedure in real samples, the levels of BPA, E2, and TT were analyzed in testis and in vFAT mass, here used as steroidogenic tissues, targeted by BPA. Indeed, the male gonadal tissue is the primary site of E2 and TT synthesis [47]. Estrogens are also synthesized in extra-gonadal sites, such as adipose tissue [48], which preferentially accumulates lipophilic chemicals, as BPA [49]. Results show that levels of BPA, E2, and TT were included in a very wide range, in both the examined tissues. For each sample (CTRL1-4 and BPA1-4), values (i.e., mean of three separate repeats ± S.D.) were expressed in ng for g of tissue (ng g^−1^) and reported in Table 4.

In testis from CTRL animals, BPA levels were detected below the detection limit (LOD). Such a samples showed E2 levels below the LOD, except one (1.56 ng g^−1^), while TT levels were really high, since these varied in a range from 215.96 to 348.57 ng g^−1^. In testis from BPA exposed mice, BPA levels ranged from 2.12 to 13.37 ng g^−1^. These samples had the highest E2 levels (from 22.56 to 228.77 ng g^−1^) and the lowest TT levels (from 36.76 to 83.45 ng g^−1^), showing that high BPA levels are related to high E2 and low TT levels.

A measurable amount of BPA, E2 and TT were detected in all the vFAT samples, also in samples from unexposed mice highlighting the difficulties in eliminating external environmental BPA- contamination. Moreover, different BPA bioaccumulation in tissues from exposed mice, in response to same BPA exposure, confirmed the need to perform a direct biological monitoring. Only measuring the BPA concentrations in target tissue, the real BPA exposure was verified and better information about the interrelationships of exposure, dose, and health effects can be provided. Differences in exposure at the equivalent dose, coupled with variations in individual susceptibility, introduce a large measure of uncertainty in the health risk assessment.

**Figure 1:**
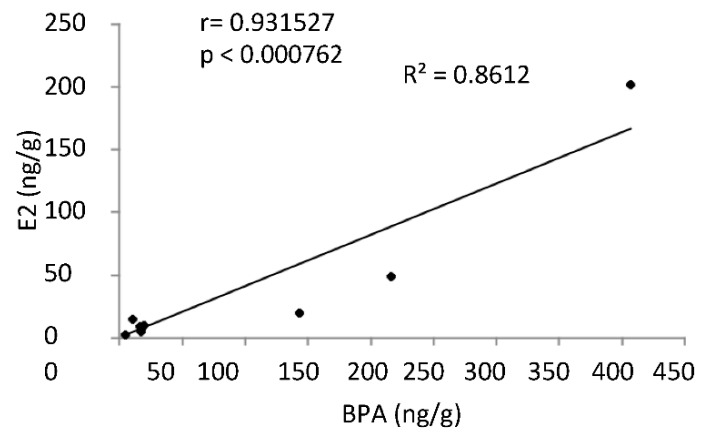
Correlation analysis between BPA and E2 levels (as ng/g) in vFAT of male mice early-life exposed to vehicle (*n* = 4) or BPA (*n* = 4) and sacrificed at 78 *dpp*. (r = 0.9315; *p* < 0.001).

Therefore, in vFat tissues, the values ranged from 4.5 to 406 ng g^−1^ for BPA, from 2.46 to 201.53 ng g^−1^ for E2, and from 6.85 to 134.45 for TT. Three of these samples had much higher BPA and E2 levels than the other ones (on average about 12 and 20 times higher for E2 and BPA, respectively) showing that high BPA levels are related to high E2 levels. The correlation analysis confirmed that BPA and E2 levels were significantly and directly related each other (Figure 1).

In agreement with the literature [18,19,20], the data reported above replicate the findings that BPA preferentially accumulates in fat tissue and also suggest that such bioaccumulation is related to steroid levels (see BPA vs. steroid amounts in each sample). Importantly, it has been demonstrated TT/E2 ratio modulate (patho)physiology of several tissues, including testis [50,51,52], emphasizing significance of the method here developed. Interestingly, our animals were exposed to BPA during the fetal-perinatal period and the three analytes, including BPA, were extracted and analyzed in adulthood animals, highlighting the occurrence of BPA bioaccumulation [53]. Furthermore, the exposure was carried out primarily via pregnant/nursing mothers so that higher BPA levels detected in exposed mice agrees with previous findings that placenta [54] and breast milk [55] are important exposure routes.

## 3. Materials and Methods

### 3.1. Materials

Bisphenol A (BPA), BPA-d_16_, 17β-estradiol (E2) and Testosterone (TT) were procured from Sigma Aldrich (Merck KGaA, Germany). HPLC grade reagents, including ultrapure water, methanol (MeOH) and acetonitrile (ACN) were acquired from Carlo Erba Reagents (Milano, Italy).

Frozen testis and vFAT mass from 3 mice were shipped in glassware and stored at −20 °C. These tissues were used only for preparation of quality control samples during the validation of the method.

### 3.2. Preparation of Standard Reference Materials and Control Samples

Standard stock solutions of a single analyte at 1.0 mg/mL were prepared in MeOH. Similarly, stock standard solution of BPA-d_16_, used as internal standard (IS) within each sample to monitor method performance, was prepared at 1 mg/mL in MeOH. Standard stock solutions were stored in dark glass vials at −20 °C until their use. To generate calibration curves, the mixture of standard solutions at intermediate concentrations was prepared from the stock solutions by serial dilution with MeOH/water (1:1, *v*/*v*). These mixtures were also used to prepared control samples (QCs) at three concentration levels, namely low 1 ng/mL (L-QC), medium 10 ng/mL (M-QC), and high 50 ng/mL (H-QC), chosen according to the endogenous levels for the hormones in reference samples. Briefly, fixed mg of homogenized target tissue (testis and vFAT from

3 mice chosen as reference materials) were spiked by adding 100 uL of mixture standard solution (at suitable concentration) and then extracted following procedures below.

### 3.3. Experimental Design and Sample Collection

Animals were exposed to BPA during fetal perinatal period as previously reported [56], and analyzed in adulthood, at 78 days post-partum (dpp). Briefly, CD1 male mice were exposed to drinking water containing ethanol alone (0.2% as vehicle; *n* = 4, unexposed/control group; CTRL) or BPA (10 µg/mL BPA dissolved in 0.2% ethanol; *n* = 4, exposed group), via pregnant/nursing mothers [from 10 days post coitum (*dpc*) to 31 *dpp*] o via direct access to water (from 21-to-31 *dpp*).

At 78 *dpp* all the animals were sacrificed by cervical dislocation under anesthesia and subjected to tissue collection. Specifically, testis and vFAT mass were dissected [56,57], properly stored at −80 °C and later processed. 

The experimental design was structured to exclude any environmental BPA-contamination as already described [56]. Experiments were approved by the Italian Ministry of Education and the Italian Ministry of Health. Procedures involving animal care were carried out in accordance with National Research Council’s publication Guide for Care and Use of Laboratory Animals (NationalInstitutes of Health Guide).

### 3.4. Extraction and Clean Up of Visceral Fat Mass

Centrifugation at 3500 rpm and 24 °C was conducted for 10 min. The supernatants were dried under a gentle stream of nitrogen gas at room temperature. The residues were dissolved with 2 mL of MeOH, stirred (5 min) at room temperature and diluted with HPLC grade water 1:1 (*v*/*v*). After loaded onto steroids specific SPE cartridges (AFFINIMIP, Polyntell SA, Paris, France) according to the manufacturer instructions, each sample was eluted with 3 mL MeOH and subsequently concentrated under a stream of nitrogen gas at room temperature to a final volume of approximately 0.5 mL. The sample was diluted to 1.0 mL final volume with HPLC grade water.

The extraction procedures were monitored using BPA-d_16_ as internal standards (IS). All resulting concentration data in samples were corrected on the basis of recovery percentage of the surrogate.

### 3.5. Extraction and Clean up of Testis

An aliquot of testicular tissue (20 ± 3 mg) was added to MeOH 1:6 (*w/v*), containing BPA-d16 as IS (100 μL at 1 μg/mL). Sample was homogenized (3 min at 15,000 rpm) by using a Diax 900 homogenizer (Heidolph Instruments Gmbh & Co.KG, Schwabach, Germany), stirred (5 min) at room temperature and then centrifuged (10 min at 3500 rpm) at 24 °C. The supernatant was recovered and diluted with HPLC grade water 1:1 (*v*/*v*). A solid-phase extraction (SPE) was performed on steroids specific AFFINIMIP for simultaneous extraction of the analytes of interest, following the procedure used for vFAT tissues.

### 3.6. Instruments, Chromatographic and Mass Spectrometry/MS Conditions

The instrumental analysis was conducted by a Dionex UltiMate3000 HPLC system (Thermo Fisher Scientific Inc, Rodano, Italy). The chromatographic separation was carried on a Phenomenex Kinetex F5 100A (100 mm × 3.0 mm, 2.6 um) with a Phenomenex SecurityGuard 4 × 2.00 mm with a mobile phase containing water (solvent A) and methanol (solvent B), under linear gradient elution. 

The gradient program started with 30% methanol for 2 min and changed linearly to 95% in 6 min, then held at 95% for 2 min. The mobile phase B was then decreased from 95% to 30% in 1 min, and finally held at 30% for 2 min to obtain a total run time of 15 min. The flow rate was 0.3 mL/min. 

Mass spectrometer was a triple quadrupole instrument (API 2000; Sciex, Milano, Italy) equipped with a TurboIon electrospray source, operating at 450 °C and at ion electrospray voltage of 5.0 kV or −4.5 kV. The Analyst™ software (Sciex, Milano, Italy) enabled to data acquisition, processing, analysis, and reporting.

### 3.7. Method Validation

The method was validated for linearity, selectivity and specificity, efficiency, accuracy, and precision according to the guidelines for bioanalytical methods established by Food and Drug Administration [58].

#### 3.7.1. Linearity

Calibration curves were determined by analyzing extracts of target tissue fortified with a known amount of each analyte as well as IS. Linear calibration curves for the three analytes were prepared plotting by least-squares regression of the analytical signal (peak area) versus nominal concentration. Linearity was estimated by the coefficient of correlation (R^2^ > 0.98). The unknown concentrations of the samples were obtained by interpolation on such curves.

#### 3.7.2. Limits of Detection (LOD) and Quantification (LOQ)

The limits of detection and quantification, relative to a single analyte, were calculated by using signal-to-noise method. The peak-to-peak noise around the single analyte retention time was measured, and subsequently, the concentration of the analyte that yielded a signal-to-noise ratio (S/N) of 3 was accepted for estimating LOD and signal-to-noise ratio of ten was used forestimating LOQ.

#### 3.7.3. Process Efficiency

Three replicates of quality control samples (1.0, 10.0, and 50.0 ng/mL) were prepared following the procedure reported in Section 2.2, extracted and analyzed. To calculate process efficiency, the peak area of the single analyte was compared with the peak area relative to the same concentration of unextracted standard.

#### 3.7.4. Precision

Precision was studied at two levels, repeatability and reproducibility. The repeatability (intra-day precision) was done by quantifying in five replicates (*n* = 5) the concentration of control samples at three different levels (1.0, 10.0 and 50.0 ng/mL) during the same day. The reproducibility (inter-day precision) was determined by measuring the concentration of the same control samples over three different days, with two different analysts and with a daily-prepared standard curve, in five replicates. The intra-day and inter-day precisions were evaluated in terms of the relative standard deviation (%RSD) that must be lower than 18%.

#### 3.7.5. Interference and Carryover

In order to verify that the instrument baseline was zero, and to monitor sensitivity changes, one or more blank samples were injected randomly during each set of samples, while the standard solutions for the calibration curve were injected at the end of the sample analyses. Furthermore, at each work session, procedural blanks, which were processed and handled similarly to the actual samples, were used for monitoring interference and avoiding cross contaminations. If traces of analytes were detected in method blanks, the values were subtracted from sample concentrations.

## 4. Conclusions

The proposed study describes an accurate and sensitive LC-MS/MS assay for the simultaneous measurement of BPA, E2, and TT in two tissues: testis and vFAT mass. The validated method is resulted reliable, sensitive, accurate, and precise. The use of specific SPE cartridges for affinity chromatography purification allows obtaining high percentages of process efficiency. The high sensitivity of tandem mass spectrometry determination allows for the quantification of BPA, E2 and TT in a single assay, even at low levels, with important biological implications as it reveals their relative amount in each sample.

As far as we know, this is the first method useful to efficiently and simultaneously extract the three analytes from testis and vFat. It can thus be used not only in studies in the reproductive field, but also in studies investigating the complex framework of metabolic syndrome.

## Figures and Tables

**Table 1:** Retention time, ions/transitions selected, and multiple reaction monitoring (MRM) conditions.

Analyte	RT (min)	ESI (kV)	Precursor Ions (*m/z*)	Product Ions ^a^ (*m/z*)	DP (V)	EP (V)	CE (eV)	CXP (V)
BPA	7.3	−4.5	227.1	212.1, 133.0	−40	−10	−25	−8
BPA-d_16_	7.3	−4.5	243.1	215.0, 138.2	−60	−7	−35	−10
17β-Estradiol	8.0	−4.5	271.0	145.0, 183.0	−70	−10	−55	−8
Testosterone	8.6	5.0	288.9	97.1, 109.2	90	7	40	10

RT: Retention time; ESI: ElectroSpray Ionization; DP: Declustering Potential; EP: Entrance Potential; CE: Collision Energy; CXP: Collision Exit Potential. ^a^ Underlined value: ion transition selected for quantification purposes.

**Table 2:** Validation data including method limits of detection (LOD) and quantification (LOQ), calibration curve equation, R2.

Matrix	Analyte	LOD (ng/mL)	LOQ (ng/mL)	Equation	R^2^
testis	BPA	0.03	0.12	*y =* 60 *+* 565*x*	0.998
	E2	0.03	0.12	*y =* 68.5 *+* 373*x*	0.997
	TT	0.05	0.18	*y =* 98.6 *+* 628*x*	0.994
vFAT	BPA	0.04	0.12	*y =* 40.2 *+* 629*x*	0.992
	E2	0.06	0.2	*y =* 74.7 *+* 388*x*	0.996
	TT	0.08	0.3	*y =* 89.7 *+* 703*x*	0.991
matrix-free(water/MeOH)	BPA	0.03	0.1	*y =* 55.9 *+* 593*x*	0.999
	E2	0.05	0.15	*y =* 81.0 *+* 379*x*	0.997
	TT	0.06	0.2	*y =* 103*+* 663*x*	0.996

**Table 3:** Mean percentage process efficiency, precision and accuracy in spiked tissues.

Tissue	Analyte (ng/mL)	Process Efficiency (%)			*Intra-Day*						*Inter-Day*					
					Precision (%RDS)			Accuracy (%)			Precision (%RDS)			Accuracy (%)		
		1	10	50	1	10	50	1	10	50	1	10	50	1	10	50
testis	BPA	77.9	83.3	76.6	12.0	11.2	10.9	102.4	98.7	97.2	14.8	13.9	14.0	104.3	98.2	91.6
	E2	74.1	80.2	80.0	13.0	12.2	11.2	100.5	98.4	99.8	16.3	14.7	13.3	98.3	96.4	95.7
	TT	70.2	74.5	68.0	12.6	13.1	10.5	99.9	98.6	94.6	16.4	16.9	14.8	97.6	95.9	88.9
vFAT	BPA	66.4	68.3	69.7	12.9	11.3	11.7	101.3	97.2	95.6	15.8	14.3	12.1	94.1	94.0	90.5
	E2	69.2	67.4	67.0	13.2	12.6	10.8	103.1	97.9	88.8	15.9	15.1	16.0	90.6	92.3	85.6
	TT	65.4	63.7	70.7	14.2	13.4	12.6	99.4	93.6	91.5	17.1	14.5	17.2	91.0	99.1	97.3

**Table 4:** BPA, TT, and E2 content (ng/g) in testis visceral fat (vFAT) of male mice early-life exposed to vehicle (CTRL1-4) or BPA (BPA1-4). TT: testosterone; E2: 17β-Estradiol.

*Testis*				*vFat*		
	BPA (ng/g)	E2 (ng/g)	TT (ng/g)	BPA (ng/g)	E2 (ng/g)	TT(ng/g)
CTRL1	<LOD	<LOD	227.42 ± 2.22	19.44 ± 0.76	9.25 ± 0.6	6.85 ± 0.59
CTRL2	<LOD	1.56 ± 0.12	215.96 ± 3.10	16.97 ± 1.24	4.35 ± 0.51	44.47 ± 1.55
CTRL3	<LOD	<LOD	318.72 ± 0.98	16.18 ± 0.75	8.63 ± 0.73	89.50 ± 1.60
CTRL4	<LOD	<LOD	348.57 ± 1.89	10.25 ± 0.72	14.72 ± 0.33	134.45 ± 2.28
BPA1	10.29 ± 0.65	102.94 ± 1.56	36.76 ± 1.55	406.78 ± 2.38	201.53 ± 0.88	114.44 ± 1.66
BPA2	6.02 ± 0.32	22.56 ± 2.45	83.45 ± 0.89	216.01 ± 1.37	48.75 ± 1.12	103.44 ± 2.18
BPA3	13.37 ± 0.68	144.92 ± 1.54	37.43 ± 0.78	142.85 ± 1.27	19.83 ± 1.31	36.01 ± 0.84
BPA4	2.12 ± 0.56	228.77 ± 2.33	50.23 ± 1.85	4.51 ± 0.63	2.46 ± 0.72	17.63 ± 0.88
